# Dynamic robustness of endoreversible Carnot refrigerator working in the maximum performance per cycle time

**DOI:** 10.1038/s41598-018-30847-2

**Published:** 2018-08-23

**Authors:** Ke Lü, Wenjie Nie, Jizhou He

**Affiliations:** 1 Department of Applied Physics, East China Jiaotong University, Nanchang, 330013 China; 2 Department of Physics, Nanchang University, Nanchang, 330031 China

## Abstract

In this work, we study the dynamic robustness of an endoreversible Carnot cycle working at the maximum per-unit-time performance regime, based on the linearization technique for dynamical systems and the local stability analysis. Our analysis is focused on the endoreversible Carnot refrigerator model, which works in the maximum per-unit-time coefficient of performance. At the steady-state of the maximum performance, the expressions of the relaxation times describing the stability of the system are derived. It is found that the relaxation times in the cycle condition are the function of thermal conductances *σ*_*h*_ and *σ*_*c*_, the temperatures of the heat reservoirs *T*_*h*_ and *T*_*c*_, and the heat capacity *C*. The influence of the temperature ratio *τ* = *T*_*c*_/*T*_*h*_ and the thermal conductance ratio *σ*_*r*_ = *σ*_*h*_/*σ*_*c*_ on the relaxation times is discussed in detail. The results obtained here are useful and provide a potential guidance for the design of an endoreversible Carnot refrigerator working in the maximum performance per cycle time optimization condition.

## Introduction

In the past years, the finite-time thermodynamics (FTT) has attracted much attentions as it is an extension of traditional equilibrium thermodynamics and used for obtaining more realistic limits for the performance of real heat devices, especially the heat engines^1–4^. The main goal of FTT is to ascertain the best operating mode of heat devices with the real-like features or the finite-time cycles. Basically, the finite-rate constrains arising from several internal and external sources of irreversibility are modeled and then the suitable objection functions, i.e., the efficiency, the power output, the coefficient of performance, the cooling power, the ecological function and so on, are optimized with respect to the involved system parameters. In addition, other thermodynamic optimization models, such as, the organic rankine cycle converting a low grade thermal energy to mechanical work, have been widely investigated by considering the exergy destructions of the system components^5–10^. The effects of the heat transfer roadmaps and the integration temperature difference on the thermodynamic property of the thermal engines have been analyzed in detail^11,12^.

In finite-time thermodynamics, the simplest and most extensively studied FTT system is the so-called edoreversible Curzon-Ahborn-Novikon (CAN) engine^3,13^. Its efficiency at maximum power output is given by ${\eta }_{CAN}=1-\sqrt{\tau }$ (where *τ* = *T*_*c*_/*T*_*h*_ is the ratio between the cold and hot reservoir temperatures). The remarkable result provides a simple and more realistic alternative expression to the Carnot efficiency (*η* = 1 − *τ*), giving a much better agreement with observed values in real power plants^14^. In this model, the only source of irreversibility is the coupling between the working substance and the heat reservoirs, and through heat conductors governed by the Newton’s heat transfer law. However, in real engines not all heat transfers obey this law. Therefore, it is essential to study the effect of different heat transfer laws. This issue has been extensively studied by several authors^15–19^. FTT theory is also used for analyzing the performance characteristics of a refrigerator, although the results attained are less satisfactory than for heat engines^20–22^. On the basis of these works, many important irreversible models of the heat engine or the refrigerator were established to assess the effect of the finite-rate heat transfer, together with other major irreversibility on the performance of cycle. The optimal performance characteristics were analyzed at the maximization of the power output and efficiency^23–25^, the minimization of the entropy generation^3,26^, the so-called ecological optimization^27,28^, and per-unit-time efficiency or coefficient of performance optimization suggested by Ma^29^ and studied in detail by Velasco and co-workers^30–32^. The similar analysis for an irreversible refrigerator was discussed by Yan and co-worker^33^.

Note that most of the studies of FTT systems have focused on their steady-state energetic properties but completely ignored their dynamic behaviors. In general, the real heat devices may deviate from the steady-state working point slightly and there exists an intrinsic cycle variability in the operation of the cycle. In other words, the dynamic robustness of the system, which is as a key property in the emerging area of constructed theory^34,35^, should be taken into account for building an energy-converting device. It allows the system to maintain its function despite internal and external perturbations in a steady-state point. Therefore, it is necessary to analyze the effect of noisy perturbations on the stability of system’s steady-state. In 2001, the study about the local stability analysis of an endoreversible Carnot engine operating under maximum power conditions is proposed firstly^36^, in order to enhance the dynamic robustness of an energy conversion system. Later, the influence of the heat transfer laws and the thermal conductance as well as the internal irreversibility of cycle on the local stability of an endoreversible heat cycle^37–40^ are studied extensively. Recently, there have been many interesting results on the stability of various energy systems working at the optimal conditions^41–45^, i.e., the heat pump working in the minimum power input or the non-endoreversible engine working in an ecological regime. In particular, the local stability analysis of a low-dissipation heat cycle working at maximum power output is discussed in detail^46^. Note that the stability of an endoreversible Carnot refrigerator working in the maximum per-unit-time coefficient of performance has not yet been discussed. In this paper, we study in detail the stability of an endoreversible refrigerator working in the maximum per-unit-time coefficient of performance^31,32^, based on the linearization technique for dynamical systems and local stability analysis. Some useful results are derived about the dynamic robustness of the endoreversible Carnot cycle system working in the optimal steady-state condition.

## Steady-State Properties of the Maximum Per-Unit-Time Performance

**Figure 1:**
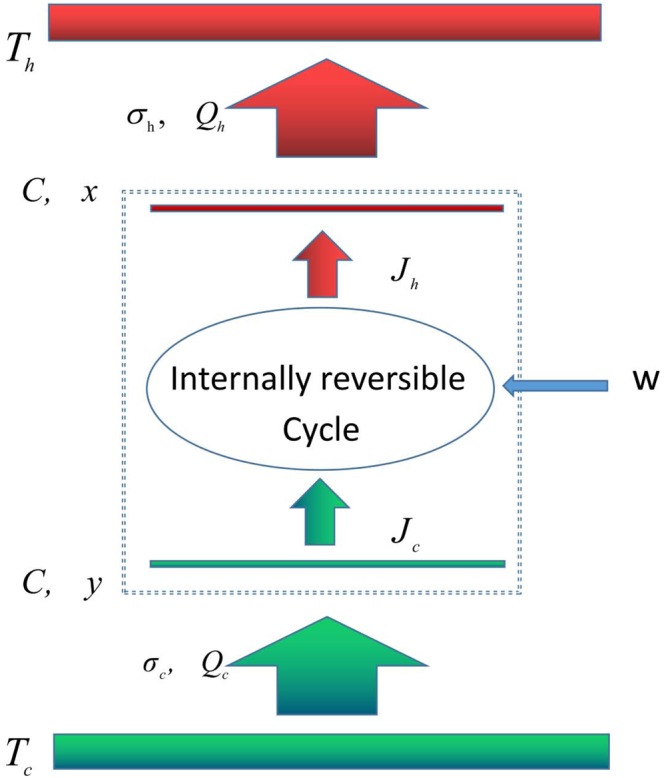
Schematic diagram of an endoreversible Carnot refrigerator (*T*_*h*_ < *x*, *y* < *T*_*c*_).

Consider a continuous endoreversible Carnot refrigerator model^30–32^ shown in Fig. 1. In the endoreversible mode, we assume that the refrigerator cycle is an internal reversible Carnot cycle working between the heat reservoirs at temperatures *x* and *y*, where *x* and *y* are, respectively, the temperatures of the working substance along the upper and the lower isothermal processes. The corresponding work input per cycle is *W*. *J*_*h*_ and *J*_*c*_ are, respectively, the heat flows from refrigerator to the reservoir *x* and from the reservoir *y* to refrigerator. Moreover, the working substance of the cycle is alternately connected to a hot reservoir at constant temperature *T*_*h*_ (*T*_*h*_ < *x*) and to a cold reservoir at constant temperature *T*_*c*_ (*T*_*c*_ > *y*). Correspondingly, *Q*_*h*_ and *Q*_*c*_ are, respectively, the heats transferred per cycle by the working substance to the hot reservoir at constant temperature *T*_*h*_ and from the cold reservoir at constant temperature *T*_*c*_. The heat transfer can be realized by the heating or cooling heat exchanger^11^. It is noted that in general a heat engine or refrigerator may contain other components such as pump or turbine and the imperfect conversion between work and energy of these components may affect the state parameters and the thermodynamic performance of the refrigerator cycle, i.e., the coupling between heat source and the working substance of the cycle^11^. Even so, in the present model we focus mainly on the local stability of the cycle system by assuming that the temperatures x and y correspond to macroscopic objects with a finite heat capacity C, and the imperfect conversion between work and energy of these components is not included. Further, from linear conduction laws (Newton’s heat transfer law) for heat transfers between the internal working substance and the external heat reservoirs, one has^30–32^

1 $${Q}_{h}={\sigma }_{h}(x-{T}_{h}){t}_{0}$$

and

2 $${Q}_{c}={\sigma }_{c}({T}_{c}-y){t}_{0}$$

where *σ*_*h*_ and *σ*_*c*_ are the external hot-end and cold-end thermal conductances, respectively, both which depend on the heat transfer area of the system^47^. The units of *σ*_*h*,*c*_ are *W*/*K*. *t*_0_ is the overall cycle time. Since the cycle is internally reversible, it verifies *Q*_*h*_/*x* = *Q*_*c*_/*y*. Then, the work input of the cycle can be calculated straightforwardly as *W* = *Q*_*h*_ − *Q*_*c*_ so that the coefficient of performance (COP) *ε* = *Q*_*c*_/(*Q*_*h*_ − *Q*_*c*_).

By optimizing the objection function, i.e., the per-unit-time COP, *ε*/*t*_0_, the optimal temperature of the working substance is^30,32^

3 $$\bar{x}/{T}_{h}=1+\sqrt{(1-\tau )/(1+{\sigma }_{r})},$$

where *σ*_*r*_ = *σ*_*h*_/*σ*_*c*_ (*σ* = *σ*_*h*_ + *σ*_*c*_ kept constant), *τ* = *T*_*c*_/*T*_*h*_, and $\bar{x}$ is the steady-state temperature corresponding to the maximum per-unit-time COP. In general, the temperature of the working substance always needs to decay the steady-state when the temperatures of the external reservoir vary. Otherwise the system may lose the stability and deviate gradually from the optimal working point. In the paper we focus mainly on the local stability of the cycle system based on the linearization technique and local stability analysis^36,37^.

Furthermore, the COP of an endoreversible refrigerator under the optimal steady-state condition is

4 $$\bar{\varepsilon }=\frac{\bar{y}}{\bar{x}-\bar{y}}=\frac{\tau }{1-\tau +\sqrt{(1-\tau )\,(1+{\sigma }_{r})}},$$

and the corresponding cooling power per cycle $\bar{R}={\dot{Q}}_{c}$ is

5 $$\bar{R}=\frac{\sigma {T}_{c}{\sigma }_{r}\sqrt{1-\tau }}{{(1+{\sigma }_{r})}^{3/2}\,[1+\sqrt{(1-\tau )(1+{\sigma }_{r})}]}.$$

**Figure 2:**
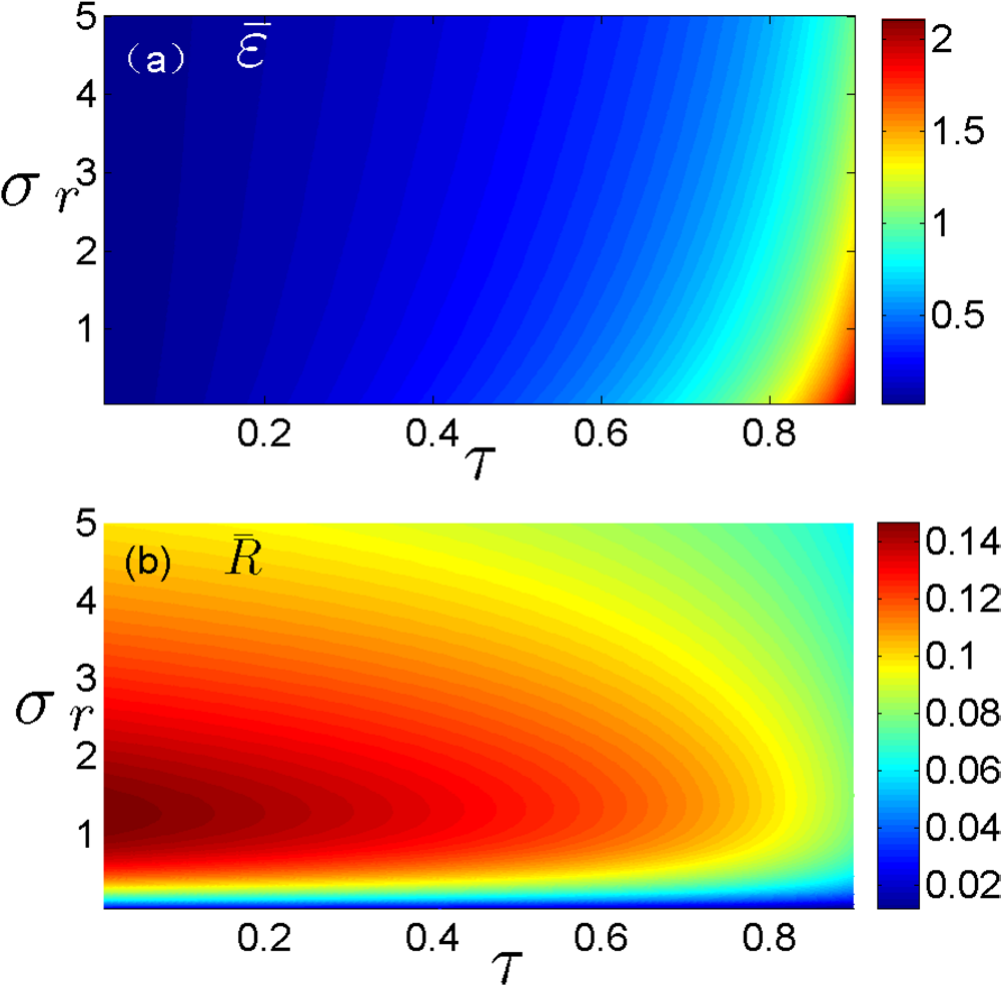
Plots of the steady-state COP and the corresponding cooling power of refrigerator per cycle versus *τ* and *σ*_*r*_ at of the maximum per-unit-time COP.

Here and thereafter, the variables with overbar represent the steady-state values corresponding to the optimization condition of the performance per cycle time, and dot represents time derivative. In Fig. 2, we show the contour plots of the steady-state COP and the corresponding cooling power versus the temperature ratio *τ* and the thermal conductance ratio *σ*_*r*_. It is seen clearly from Fig. 2 that the steady-state COP $\bar{\varepsilon }$ is monotonically increasing function of *τ*, while the steady-state cooling power $\bar{R}$ is monotonically decreasing function of *τ*. This means that although the endoreversibel refrigerator in the steady-state has high COP, the cooling power $\bar{R}$ is very small, as *τ* approaches 1.

In order to study the local stability of the endoreversible refrigerator working at the optimal condition, the steady-state cooling power per cycle should be written as a function of $\bar{x}$ and $\bar{y}$ using Eqs (3–5), i.e.,

6 $$\bar{R}(\bar{x},\bar{y})=\frac{\sigma \bar{m}\bar{x}(1-{\bar{m}}^{2})\,[1-{(1+{\sigma }_{r})}^{-1}]}{(\sqrt{1+{\sigma }_{r}}+\bar{m})\,(1+\bar{m}\sqrt{1+{\sigma }_{r}})},$$

where $\bar{m}=\sqrt{(1+{\sigma }_{r})\,({\bar{y}}^{2}{\bar{x}}^{-2}/4)-\bar{y}/\bar{x}+1}-\sqrt{1+{\sigma }_{r}}\bar{y}/2\bar{x}$. Then, under the steady-state of the maximal per-unit-time COP condition, the steady-state heat flows ${\bar{J}}_{h}$ (${\bar{J}}_{h}={\dot{Q}}_{h}$) from refrigerator to the reservoir $\bar{x}$ and ${\bar{J}}_{c}$ (${\bar{J}}_{c}={\dot{Q}}_{c}$) from the reservoir $\bar{y}$ to refrigerator can be, according to the first and second laws of thermodynamics, written as

7 $${\bar{J}}_{h}=\bar{x}\bar{R}(\bar{x},\bar{y})/\bar{y}$$

and

8 $${\bar{J}}_{c}=\bar{R}(\bar{x},\bar{y}),$$

respectively.

## Non-Steady-State Characteristics and Dynamic Equations of the Endoreversible Carnot Refrigerator

Because of the existence of the heat conductances between the working substance and the heat reservoirs, the system may deviate from the steady-state working point when the temperatures of heat reservoirs vary slightly. In particular, the temperatures of working substance depend on the time variable *t*, i.e., *x* = *x*(*t*) and *y* = *y*(*t*), so that the intrinsic cyclic variability appears in the system. This means that in the condition deviating from the steady-state, the thermodynamic relations ${J}_{h}={\dot{Q}}_{h}$ and ${J}_{c}={\dot{Q}}_{c}$ in the endoreversible cycle will not be valid. Here *J*_*h*_ and *J*_*c*_ represent the non-steady-state heat flows between the reservoirs and working substance. Santillan and co-workers^36^ have developed a system of coupled differential equations to describe the rate at which the temperature of working substance in the thermodynamic cycle is changing with respect to the independent variable *t*. Correspondingly, the stability of the system can be analyzed by assuming that the temperatures *x* and *y* corresponding to macroscopic objects with a finite heat capacity *C*. Then the change of the temperatures *x* and *y* for the present cycle model can be described as

9 $$dx/dt=({J}_{h}-{\dot{Q}}_{h})/C$$

and

10 $$dy/dt=({\dot{Q}}_{c}-{J}_{c})/C,$$

where the non-steady-state heat flows ${\dot{Q}}_{h}$ and ${\dot{Q}}_{c}$ in the differential equations are the function of the temperature *x* and *y*. The specific expressions are attained in terms of Eqs (1 and 2).

It is worth to note that we should give the specific expressions of the heat fluids in the non-steady-state condition. When the refrigerator system works out of but not too far from the steady-state, we can assume as a first approximation that Eqs (6–8) for the endoreversible refrigerator is also valid^36–41^. Then, the cooling power per cycle *R* depends on *x* and *y* in the same way as $\bar{R}$ depends on $\bar{x}$ and $\bar{y}$ at the steady-state, that is, $R(x,y)=\bar{R}(\bar{x},\bar{y})$. The purpose of the treatment is very simple to understand the intrinsic properties of machines, in line with constructed theory^34^. Therefore, using Eqs (7 and 8), *J*_*h*_ and *J*_*c*_ in terms of *x*, *y* and *R*(*x*, *y*) can be written as

11 $${J}_{h}=xR(x,y)/y$$

and

12 $${J}_{c}=R(x,y).$$

By making use of the approximation $R(x,y)=\bar{R}(\bar{x},\bar{y})$, the differential dynamic equations [Eqs (9 and 10)] for *x* and *y* can be expressed as

13 $$\frac{dx}{dt}=\frac{1}{C}\,[\frac{x}{y}R(x,y)-\frac{\sigma {\sigma }_{r}}{1+{\sigma }_{r}}(x-{T}_{h})]$$

and

14 $$\frac{dy}{dt}=\frac{1}{C}\,[\frac{\sigma ({T}_{c}-y)}{1+{\sigma }_{r}}-R(x,y)],$$

where $$R(x,y)=\tfrac{\sigma mx(1-{m}^{2})\,[1-{(1+{\sigma }_{r})}^{-1}]}{(\sqrt{1+{\sigma }_{r}}+m)\,(1+m\sqrt{1+{\sigma }_{r}})}$$ and $$m=\sqrt{(1+{\sigma }_{r})\,({y}^{2}{x}^{-2}/4)-y/x+1}-\sqrt{1+{\sigma }_{r}}y/2x$$.

## Local Stability Analysis and System Dynamic Robustness

Consider a set of the dynamical system *dx*/*dt* = *f*(*x*, *y*) and *dy*/*dt* = *g*(*x*, *y*). Its steady-state is the couples $\bar{x},\bar{y}$ that simultaneously satisfy $f(\bar{x},\bar{y})=0$ and $g(\bar{x},\bar{y})=0$. That is, if $f(x,y)=\frac{1}{C}[\frac{x}{y}R(x,y)-\frac{\sigma {\sigma }_{r}}{1+{\sigma }_{r}}(x-{T}_{h})]$ and $g(x,y)=\frac{1}{C}[\frac{\sigma ({T}_{c}-y)}{1+{\sigma }_{r}}-R(x,y)]$, then the unique steady state is given by Eqs (3 and 4) for the refrigerator cycle. In particular, following Strogatz^48^, the steady-state local stability is determined by the eigenvalues of the Jacobian matrix: $$J=(\begin{array}{cc}{f}_{x} & {f}_{y}\\ {g}_{x} & {g}_{y}\end{array})$$, where ${f}_{x}={(\frac{\partial f}{\partial x})}_{\bar{x},\bar{y}}$, ${f}_{y}={(\frac{\partial f}{\partial y})}_{\bar{x},\bar{y}}$, ${g}_{x}={(\frac{\partial g}{\partial x})}_{\bar{x},\bar{y}}$ and ${g}_{y}={(\frac{\partial g}{\partial y})}_{\bar{x},\bar{y}}$. Let us suppose that *λ*_1_ and *λ*_2_ denote the eigenvalues of Jacobian matrix and ${\overrightarrow{u}}_{1}$ and ${\overrightarrow{u}}_{2}$ the corresponding eigenvectors. The general solution $\delta \overrightarrow{r}=(\delta x,\delta y)$ of small perturbations from the steady-state, *δx* and *δy*, is given by $\delta \overrightarrow{r}(t)={c}_{1}{\overrightarrow{u}}_{1}{e}^{{\lambda }_{1}t}+{c}_{2}\overrightarrow{{u}_{2}}{e}^{{\lambda }_{2}t}$. Then, if both the eigenvalues are real and negative, the perturbations *δx* and *δy* converge to zero monotonically and the steady-state of the system is stable. Specifically, the eigenvalues *λ*_1_ and *λ*_2_ can be calculated by the characteristic equation

15 $$({f}_{x}-\lambda )\,({g}_{y}-\lambda )-{g}_{x}\,{f}_{y}=0.$$

Using Eqs (13–15) the eigenvalues for the endoreversible refrigerator cycle can be derived as

16 $${\lambda }_{1,2}=\frac{\sigma }{C}\frac{{\sigma }_{r}(\alpha \pm \sqrt{{\alpha }^{2}-4\beta })}{2(1+{\sigma }_{r})},$$

where

17 $$\alpha =-\,1-{\sigma }_{r}^{-1}+2({a}_{2}+{a}_{4})/{a}_{0},$$

18 $$\beta =(\,-\,{\sigma }_{r}^{-1}+{a}_{4}/{a}_{0})\,(\,-\,1+2{a}_{2}/{a}_{0}+{a}_{4}/{a}_{0})-{({a}_{2}+{a}_{4})}^{2}/{a}_{0}^{2},$$

19 $${a}_{0}=\tau /[1+\sqrt{(1-\tau )\,(1+{\sigma }_{r})}],$$

20 $${a}_{1}=\sqrt{(1+{\sigma }_{r}){a}_{0}^{2}/4-{a}_{0}+1}-{a}_{0}\sqrt{1+{\sigma }_{r}}/2,$$

21 $${a}_{2}={a}_{1}(1-{a}_{1}^{2})/[(\sqrt{1+{\sigma }_{r}}+{a}_{1})\,(1+{a}_{1}\sqrt{1+{\sigma }_{r}})],$$

22 $${a}_{3}=\frac{{a}_{0}\sqrt{1+{\sigma }_{r}}}{2}+\frac{1}{2}\frac{{a}_{0}-{a}_{0}^{2}(1+{\sigma }_{r})/2}{\sqrt{{a}_{0}^{2}(1+{\sigma }_{r})/4-{a}_{0}+1}},$$

23 $${a}_{4}=\tfrac{{a}_{3}(1-3{a}_{1}^{2})\,[\sqrt{1+{\sigma }_{r}}+{a}_{1}(2+{\sigma }_{r})+{a}_{1}^{2}\sqrt{1+{\sigma }_{r}}]+{a}_{3}({a}_{1}-{a}_{1}^{3})\,[(2+{\sigma }_{r})+2{a}_{1}\sqrt{1+{\sigma }_{r}}]}{{[\sqrt{1+{\sigma }_{r}}+{a}_{1}(2+{\sigma }_{r})+{a}_{1}^{2}\sqrt{1+{\sigma }_{r}}]}^{2}}.$$

One clearly sees from Eq. (16) that the eigenvalues for the endoreversible refrigerator are expressed as a function of the system parameters *C*, *σ*_*h*_, *σ*_*c*_, *T*_*h*_ and *T*_*c*_. Furthermore, with the help of the numerical solutions, it is found that both *λ*_1_ and *λ*_2_ can be real and negative for *C* > 0, *σ*_*h*_ > 0, *σ*_*c*_ > 0 and $0 < \tau \lesssim 0.75$. Thus, the steady-state of the maximum per-unit-time COP is stable and every small perturbation around the steady-state values of the temperature of the working substance would decay exponentially with time. In this case, it allows us to define relaxation times

24 $${t}_{1,2}=-\,1/{\lambda }_{1,2},$$

which describe the stability of the cycle system. In other words, the smaller the relaxation time, the better local stability of the system. Equations (16 and 24) are the main result of this paper and it gives the stability characteristics of the endoreversible Carnot refrigerator working in the maximum per-unit-time COP. The time evolution of a given perturbation from the steady state is generally determined by both the relaxation times that are also a function of *C*, *σ*_*h*_, *σ*_*c*_, *T*_*h*_ and *T*_*c*_. One may note that the relaxation times are proportional to *C*/*σ*. This means that in order to improve the systems’ stability, we should either increase *σ* or decrease *C*. Comparison with the steady-state cooling power per cycle $\bar{R}$ for the refrigerator [Eq. (5)], reveals that an increment in *σ* not only improves the system’s stability, but also increases $\bar{R}$. The characteristic is different from the previous results attained based on and endoreversible engine working in the maximum power output^36^, in which the relaxation time increases only with increasing the hot-end thermal conductances *σ*_*h*_.

**Figure 3:**
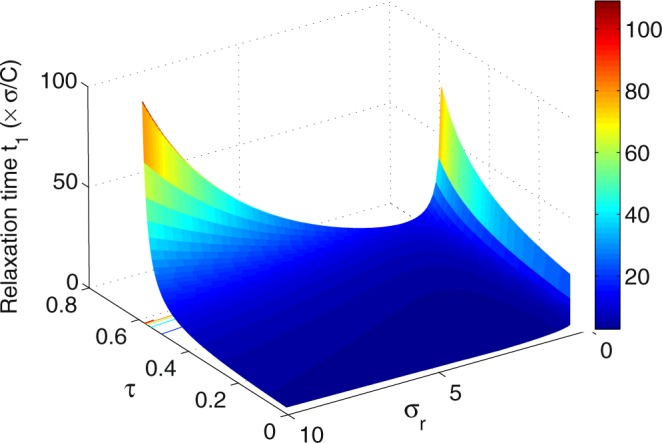
Plots of the relaxation time *t*_1_ of the refrigerator versus *τ* and *σ*_*r*_ at of the maximum per-unit-time COP.

**Figure 4:**
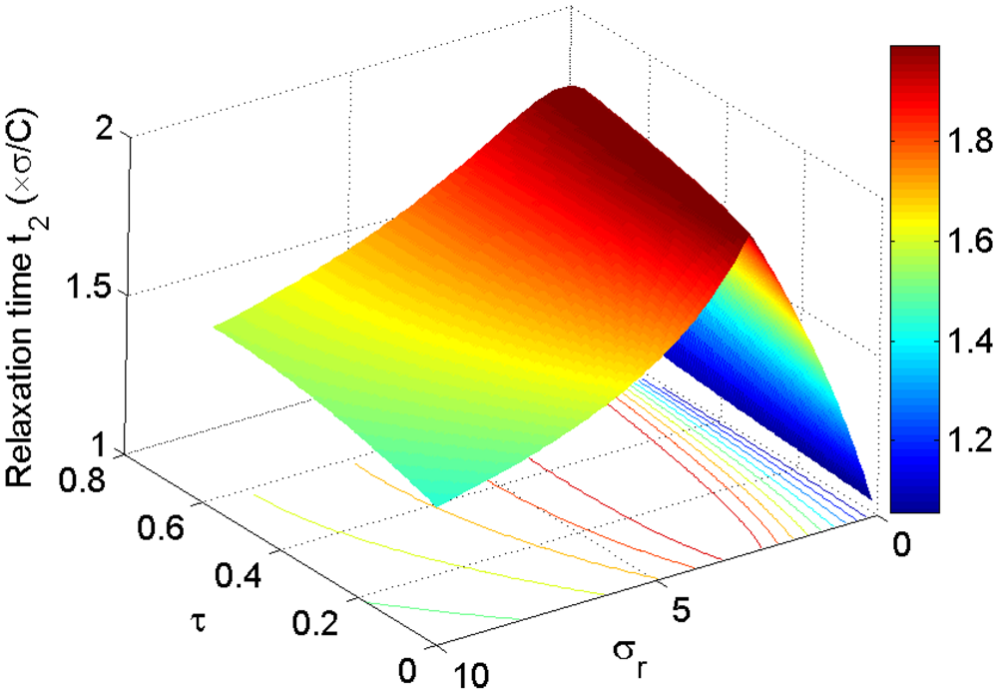
Plots of the relaxation time *t*_2_ of the refrigerator versus *τ* and *σ*_*r*_ at of the maximum per-unit-time COP.

Figure 3 shows the relaxation time *t*_1_ as a function of the temperature ratio *τ* and the thermal conductance ratio *σ*_*r*_ in the maximum per-unit-time COP of the refrigerator. It is evident that the relaxation time *t*_1_ depends strongly on *τ* and *σ*_*r*_. Further, with the different values of *τ*, the relaxation time *t*_1_ is always very large in the regime of the thermal conductance ratio approaching zero. Therefore, the stability of system declines when the values of *σ*_*r*_ approach zero. The other region of the large relaxation time *t*_1_ appears when both the temperature ratio *τ* and the thermal conductance ratio *σ*_*r*_ are large. Thus, the stability of system declines when the values of *τ* approach 1 and improves as *τ* approach 0. In contrast, the optimal value of *σ*_*r*_ corresponding to the minimum relaxation time *t*_1_ appears at the moderate values of *σ*_*r*_, in which the stability of system is enhanced. Figure 4 shows the relaxation time *t*_2_ as a function of the temperature ratio *τ* and the thermal conductance ratio *σ*_*r*_. We can see that the relaxation time *t*_2_ presents a maximum as *σ*_*r*_ ≈ 2. It is noted from Figs 3 and 4 that *t*_1_ > *t*_2_ for all the values of *τ* and *σ*_*r*_. Consequently, the decrease of relaxation time *t*_2_ is marginal for enhancing the stability of the system because the perturbation long-term behavior is dominated by the longest relaxation time, i.e., *t*_1_. By Comparing with the steady-state properties of the endoreversible refrigerator working in a maximum per-unit-time COP, the system’s stability moves in the opposite direction to that of the steady-state COP, while in the same direction to that of the steady-state cooling power per cycle, as *τ* varies. Then, the temperature ratio *τ* represents a trade-off between stability and the steady-state COP $\bar{\varepsilon }$.

**Figure 5:**
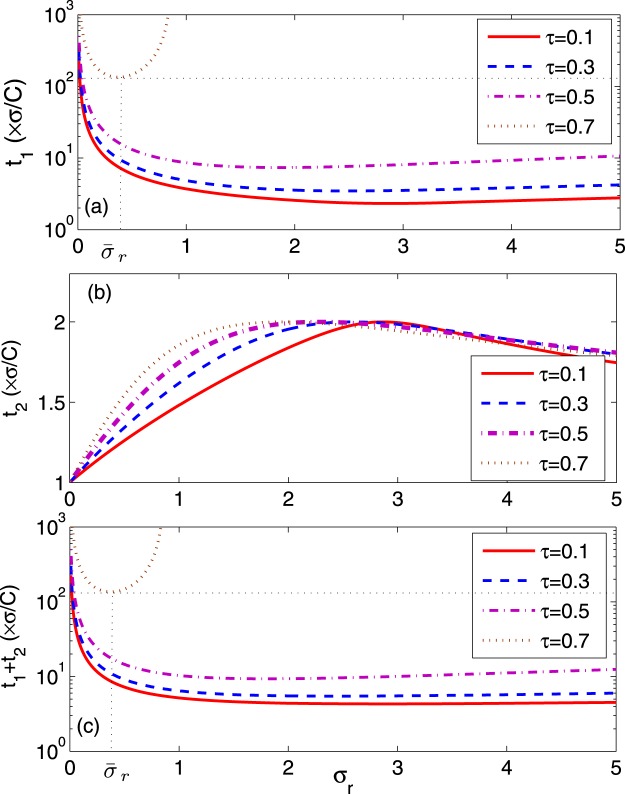
Plots of the relaxation times (**a**) *t*_1_, (**b**) *t*_2_ and (**c**) *t*_1_ + *t*_2_ versus *σ*_*r*_ with the different *τ*.

**Figure 6:**
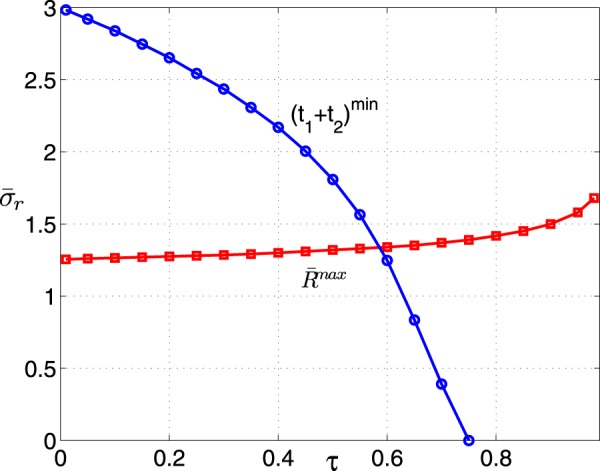
Plots of ${\bar{\sigma }}_{r}(t)$ versus *τ* at of the minimum relaxation time (*t*_1_ + *t*_2_)^*min*^ and the maximum cooling power per cycle for the endoreversible refrigerator working in the maximum per-unit-time COP.

In Fig. 5, we depicted the relaxation times *t*_1_ and *t*_2_ and the total relaxation time *t*_1_ + *t*_2_ as a function of the thermal conductance ratio *σ*_*r*_ with the different temperature ratio *τ*. Here the total relaxation time can be expressed as *t*_1_ + *t*_2_ = −*Cα*(1 + *σ*_*r*_)/(*σσ*_*r*_*β*). It is clear that the relaxation times are not monotonous function of *σ*_*r*_. Furthermore, the optimal value ${\bar{\sigma }}_{r}$ corresponding to the minimum relaxation time ${t}_{1}^{min}$ decreases with increasing *τ* [see Fig. 5(a)]. The similar behavior for the total relaxation time *t*_1_ + *t*_2_ appears as *τ* increases. In particular, we can see from Fig. 5(b,c) that the relaxation time *t*_2_ do not affect significantly total relaxation time. The plot of ${\bar{\sigma }}_{r}$ corresponding to the minimum total relaxation time (*t*_1_ + *t*_2_)^*min*^ and the maximum cooling power per cycle ${\bar{R}}^{max}$, versus *τ* is shown in Fig. 6 by the numerical calculation. It is found that from Fig. 6, under the optimal stability of system condition, i.e., (*t*_1_ + *t*_2_)^*min*^, ${\bar{\sigma }}_{r}$ decreases with increasing *τ* and is bounded by $0\lesssim {\bar{\sigma }}_{r}\lesssim 3$. While under the maximum cooling power per cycle condition ${\bar{R}}^{max}$, ${\bar{\sigma }}_{r}$ increases with increasing *τ* and is bounded by $1.25\lesssim {\bar{\sigma }}_{r}\lesssim 1.70$. Although the two optimal ranges exist superposition, it is of that the different *τ*. That is, in general for a given *τ*, the optimal values ${\bar{\sigma }}_{r}$ corresponding to the good stability of system and the maximum cooling power per cycle deviate each other. Therefore, in these cases the parameter *σ*_*r*_ represents a trade-off between the stability of the system and the optimal cooling power $\bar{R}$. In particular, as *τ* ≈ 0.6, there exists a useful optimal value ${\bar{\sigma }}_{r}=1.34$, in which the best operation mode of refrigerator including the optimal cooling power per cycle and the dynamic stability of system, can be reached. The results attained here provide a potential guidance for designing a refrigerator working in the maximum per-unit time COP. Finally, we stress that the present treatment can be also applied to an endoreversible Carnot engine working in the maximum per-unit-time efficiency or other irreversible Carnot refrigerator including internal dissipations of the working substance and heat leak between reservoirs. And then, the influences of the internal irreversibility and heat leak rate on the stability of irreversible Carnot cycle system working in the maximum performance per-unit-time can be discussed in detail. It is noted that the maximum per-unit-time efficiency of an irreversible Carnot engine cycle agrees with observed values for real power plants^30^. Thus the local stability analysis of heat engines working in the optimal steady state may be important from the point of view of design, which leads to select properly the external hot-end and cold-end thermal conductances or the heat transfer area of the thermal engine system. Furthermore, the stability analysis has many potential applications for practical engineering, i.e., the energy saving and the gas emission reduction since the heat engine has wide applications for power plants.

## Discussion

In conclusion, this work presented a local stability analysis of an endoreversible Carnot refrigerator working in the maximum per-unit-time COP. Under the maximum performance regime, the general expressions of relaxation times are derived in detail, which are shown as the function of the thermal conductances of the system, *σ*_*h*_ and *σ*_*c*_, the temperatures *T*_*h*_ and *T*_*c*_, and the heat capacity *C*. Further, it is found that the system working in the maximum per-unit-time COP can be stable because the system exponentially decays to the steady state after a small perturbation. In particular, we also discuss in detail the trade-off between the stability of system and the steady-state energetic properties by some representative examples for the endoreversible refrigerator. The results obtained here are useful for both determining the optimal operating conditions and designing of Carnot refrigerators.
